# Short-term and long-term unintended impacts of a pilot reform on Beijing’s zero markup drug policy: a propensity score-matched study

**DOI:** 10.1186/s12913-019-4764-z

**Published:** 2019-11-29

**Authors:** Jianying Zeng, Xiwen Chen, Hongqiao Fu, Ming Lu, Weiyan Jian

**Affiliations:** 1 Department of Health Policy and Management, School of Public Health, Peking University Health Science Center, Beijing, China; 2 Department of Medical Informatics, School of Basic Medicine, Peking University Health Science Center, Beijing, China

**Keywords:** Healthcare expenditure, Healthcare reform, Cost containment

## Abstract

**Background:**

In September 2012, Beijing, the capital of China, selected five tertiary hospitals as pilots to remove the previously allowed 15% markup for drug sales. However, while most research demonstrated the significant decrease in drug sales, the core issue of high health expenditure was not well solved because of the unintended policy impact. This study aimed to empirically evaluate the short-term and long-term unintended impacts on controlling medical expenses of Beijing’s zero markup drug policy from 2012 to 2015.

**Methods:**

This study extracted 2012–2015 individual-level data from the Beijing Urban Employee Basic Medical Insurance (UEBMI) database and performed a propensity score-matched analysis to evaluate the short-term and long-term impacts on controlling medical expenses. All inpatients in the 5 pilot reform hospitals were selected as the intervention group, while inpatients in other tertiary hospitals were selected as the control group.

**Results:**

A total of 520,996 inpatients were extracted in this study. For patients in the pilot hospitals, the total expenditures per admission decreased from 17,140.3 yuan in 2012 to 15,430.1 yuan in 2013 and then increased to 16,789.8 yuan in 2015. Expenditure on drugs reduced from 5811.7 yuan in 2012 to 3903.4 yuan in 2015. However, a significant substitution effect of medical consumables was first observed in the third quarter of 2014, which undermined the impact of the policy. In the long-term, the intervention group and control group demonstrated the same trend.

**Conclusions:**

After the zero markup drug policy, expenditure on drugs revealed a continuous decline. However, the decline in total expenditure was weakened by the substitution effect of medical consumables in the long term.

## Background

Over-prescription of expensive pharmaceuticals has become a global concern for increasing total health expenditure (1, 2). Many countries have implemented several policies in order to reduce drug prices and control the total growth of health expenditures. Recently, in OECD countries, drug expenditure, on average, accounted for approximately 20% of total health expenditure. In France, the cost of drugs also accounted for 17.5% of total health expenditure, while in the United States, it accounted for approximately 10% (3–5). However, compared to developed countries, China must address the greater problem of the widespread over-prescription of drugs caused by a 15% markup policy for drug sales (6). Hospitals in China can make profits from drug sales, which is one of the three main channels for the income of public hospitals. The other two income channels are medical service charges and government financial subsidies (7). Moreover, with the reductions in the government’s financial subsidies and restrictions on the price of medical services, drug expenditure has been growing and accounted for 30–40% of the total health expenditure in the 2000s (8, 9). Meanwhile, researchers have noted that the incentives from the 15% markup policy for drug sales have prompted doctors to prescribe large amounts of unnecessary antibiotics, injections and hormones such as the steroid drugs, which has become a major problem in China’s public health field (10–12).

China has begun to eliminate drug markups in the last decade but had to face the complex compensation mechanism in public hospitals (13, 14). Public hospitals in China are the dominant provider in the Chinese health system and treat more than 80% of inpatients (15). The Chinese government therefore tried to remove the previous 15% markup for drug sales at public hospitals and replaced it with the zero-markup drug policy, hoping that public hospitals could no longer make a profit from selling drugs and, predictably, irrational drug use would be reduced. In September 2012, Beijing, the capital of China, selected five tertiary hospitals as pilots to remove the previously allowed 15% markup for drug sales and implement the zero markup drug policy. And after that, the State Council of China announced that all urban public hospitals must implement the zero markup drug policy by September 2017. While most research demonstrated the significant decrease in drug sales, the core issue of high health expenditure, however, was not well solved because of the unintended policy impact. The unintended outcome of medical consumables would be even worse if the hospitals treated more inpatients and faced more administrative challenges (16), which reignited the debate about eliminating drug markups.

To date, studies on the unintended impact of the zero markup drug policy were mostly aggregated in the substitution effect of medical consumables and diagnostic tests (17), but their results were varied (18, 19). One study that analysed county-level public hospitals in Shandong province in China noted that no expenditure transfer from drugs to medical consumables or diagnostic tests was observed due to the removal of drug markups (20) while others presented the opposite case. Moreover, their reforms were multiple and complex which included not only one drug policy but varied internal reforms in hospitals. In addition, the substitution effect were less likely observed with only short-term analysis such as interrupted time series analysis because providers’ behaviours also need time to change. Thanksfully, there did exist one single reform of drug policy if back to the first pilot reform in Beijing which reformed five tertiary hospitals while other tertiary hospitals implemented the previous drug policy, providing a chance of long-term comparison.

This study aimed to evaluate the short-term and long-term impacts on medical expenses of Beijing’s zero markup drug policy from 2012 to 2015. We hypothesize that, compared with hospitals in the control group, the total expenditure of pilot hospitals would decrease in short-term thanks to the decline in drug sale but would gradually rise due to the increase of the expenditures on medical consumables and diagnostic tests.

## Methods

### Study design and participants

This study extracted four years of individual-level data from the Beijing Urban Employee Basic Medical Insurance (UEBMI) database, which has collected all inpatient medical records in Beijing public hospitals. The inpatient medical records in this study are age, gender, admission time, the main diagnosis and its ICD coding, the secondary diagnosis and its ICD coding, total hospital expenditures and the expenditure breakdown. Based on Chinese government documents and UEBMI database, hospital expenditures fall into six categories: drugs, medical services, medical consumables, diagnostic tests, nursing services and management services. Descriptions of the basic characteristics for each year and the top 10 diagnoses are listed in Table 1. All expenditure variables are converted to 2012 using the Consumer Price Index (see in the Appendix 1).

**Table 1:** Description of basic characteristics of the pilot hospitals for each year

		2012	2013	2014	2015
N	=	55,563	69,760	66,559	68,616
Age	Mean (years)	61.5	60.7	60.5	60.1
Gender	Female	25,934	33,980	31,187	32,854
	Male	29,629	35,780	35,372	35,762
CCI	0	28,008	36,670	30,149	30,458
	1	12,081	12,547	13,006	12,799
	≥2	15,474	20,543	23,404	25,359
Top 10 diagnoses	Chemotherapy session for neoplasm	8715	9644	8976	9100
	Unstable angina	3319	3283	3849	3885
	Cerebral infarction, unspecified	3424	3122	3027	3032
	Senile cataract, unspecified	1831	4229	1775	1742
	Type 2 diabetes mellitus with other specified complications	583	1880	2070	2311
	Atherosclerotic heart disease	1172	1655	1421	1473
	Unilateral or unspecified inguinal hernia, without obstruction or gangrene	1353	1380	1379	1411
	Essential (primary) hypertension	1317	1417	1398	1274
	Palliative care	1033	1414	1445	1155
	COPD with acute exacerbation, unspecified	1549	1229	1220	938

In this study, we selected all UEBMI inpatients from January 1st, 2012 to December 31st, 2015 in Beijing. In September 2012, Beijing started the single drug policy reform and at the end of 2012, all pilot hospitals accomplished zero markup drug reform. From 2016 to 2017, Beijing implemented more complex health reforms which shocked the existing medical expenses and made it hard to follow up.

Overall, a total of 1,521,659 cases from 25 large tertiary hospitals were included, and 302,005 UEBMI inpatients in the 5 pilot reform hospitals were selected as the intervention group, while 705,220 inpatients in other tertiary hospitals were selected as the control group. After exclusion of unqualified samples and 1:1 matching, the final sample size for the next analysis was 520,996.

### Propensity score matching (PSM)

**Fig. 1:**
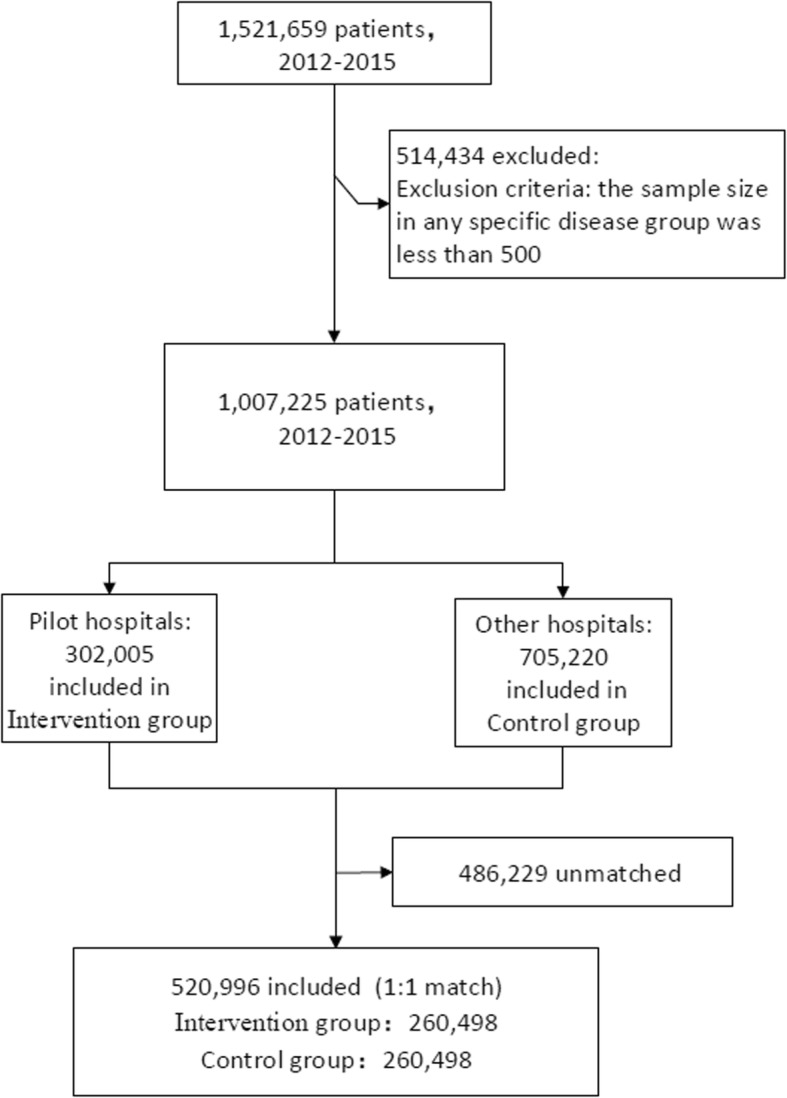
The flowchart of propensity score-matching

This study performed a 1:1 propensity score-matched analysis stratified by disease groups and years while controlling for age, gender and Charlson Comorbidity Index (CCI). Propensity scores were calculated by logistic regression. The disease groups were defined as the first four International Classification of Diseases tenth version (ICD-10) codes, the smallest unit in this coding category. CCI was calculated according to all secondary diagnoses. In this study, two exclusion criteria were chosen to ensure a smooth match process: first, the sample size in any specific disease group was less than 500; second, only the intervention group or control group were included in any specific disease group. The matching flowchart is shown in Fig. 1.

## Results

### Description of basic characteristics of the pilot hospitals

A total of 520,996 patients were included in this study, with 260,498 patients each included in each of group (the intervention group and the control group). The characteristics of the pilot hospitals and the top 10 disease groups are listed in Table 1.

### Annual changes in hospitalization expenses for pilot hospitals

The total expenditure and other expenditure components of the pilot hospitals are shown in Table 2.

**Table 2:** Annual hospitalization expenses and details for pilot hospitals

		2012	2013	2014	2015
		Treatment	Treatment	Treatment	Treatment
N	=	55,563	69,760	66,559	68,616
Total expenditures per visit		17,140.3	15,430.1	16,650.4	16,789.8
OOP		4135.5	4035.2	4349.0	4571.1
Drugs	per admission (¥)	5811.7	4304.0	4273.6	3903.4
	Share (%)	33.91%	27.89%	25.67%	23.25%
Medical services	per admission (¥)	1912.4	2213.5	2386.8	2365.5
	Share (%)	11.16%	14.35%	14.33%	14.09%
Medical consumables	per admission (¥)	6033.7	5889.0	6528.5	6966.3
	Share (%)	35.20%	38.17%	39.21%	41.49%
Diagnostic tests	per admission (¥)	2802.2	2534.2	2924.0	3025.1
	Share (%)	16.35%	16.42%	17.56%	18.02%
Nursing services	per admission (¥)	79.4	66.7	69.2	66.1
	Share (%)	0.46%	0.43%	0.42%	0.39%
Management services	per admission (¥)	500.9	422.7	468.4	463.4
	Share (%)	2.92%	2.74%	2.81%	2.76%

*Share: share in total expenditures per admission

After the zero markup drug policy, for patients in the pilot hospitals, the total expenditures per admission and out-of-pocket costs decreased in year one and increased gradually over the next few years. The total expenditures per admission decreased from 17,140.3 yuan in 2012 to 15,430.1 yuan in 2013 and then increased to 16,789.8 yuan in 2015, which was still lower than that before the policy. Out-of-pocket costs demonstrated a similar trend, which went from 4135.5 yuan in 2012 down to 4035.2 yuan in 2013 and then up to 4571.1 yuan in 2015.

Expenditure on drugs revealed a continuous decline over the next four years, while expenditure on medical services had increased growth. Expenditure on drugs reduced from 5811.7 yuan in 2012 to 3903.4 yuan in 2015, and drug expenditure as a share of total expenses per admission decreased from 33.91 to 23.25%. In contrast, expenditure on medical services rose from 1912.4 yuan in 2012 to 2365.5 yuan in 2015, and medical services expenditure as a share of total expenses increased from 11.16 to 14.09%.

Expenditure on medical consumables and diagnostic tests demonstrated the same trend of continuous rise. From 2012 to 2015, expenditure on medical consumables increased from 6033.7 yuan to 6966.3 yuan, an increase 35.20 to 41.49% of total expenses, and expenditure on diagnostic tests also increased from 2802.2 yuan to 3025.1 yuan, an increase from 16.35 to 18.02% of total expenses.

### Policy impact analysis

In order to further verify that the changes in expenditure were caused by the impact of the zero markup drug policy, we made a trend comparison with the control group. Since the pilot reform was carried out from September to December 2012 in Beijing, we chose the mean expenditure of the first three quarters of 2012 as the baseline and compared this baseline with the expenditure of every quarter after that (see more in the Appendix 2).

**Fig. 2:**
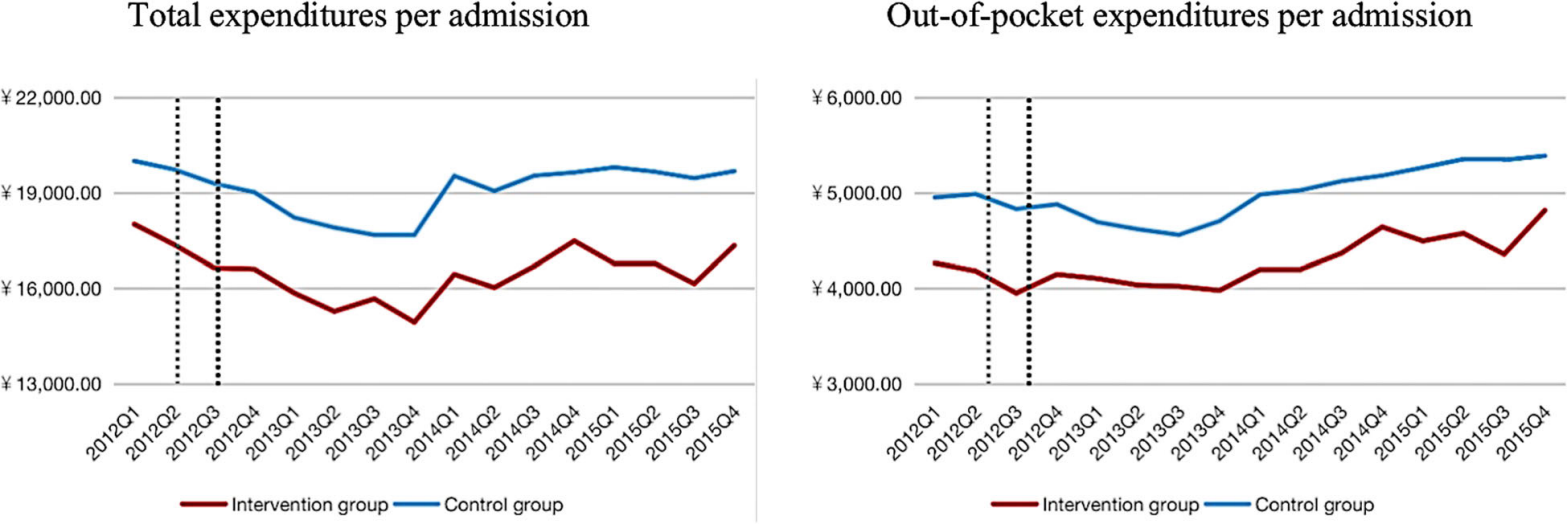
A trend comparison of total expenditures and out-of-pocket expenditures per admission between the intervention group and the control group

As for total expenditure and out-of-pocket costs, although those expenses decreased at first, the intervention group and control group showed the same trend both in the short-term and the long-term, which indicated that there was no obvious impact of the policy (Fig. 2). To explain why the trend of total expenditure did not change, we further breakdown the expenditure and carried out a specific analysis to see whether the substitution effect of medical consumables or diagnostic tests led to no significant change in the total expenditure in the long- and short-term.

**Fig. 3:**
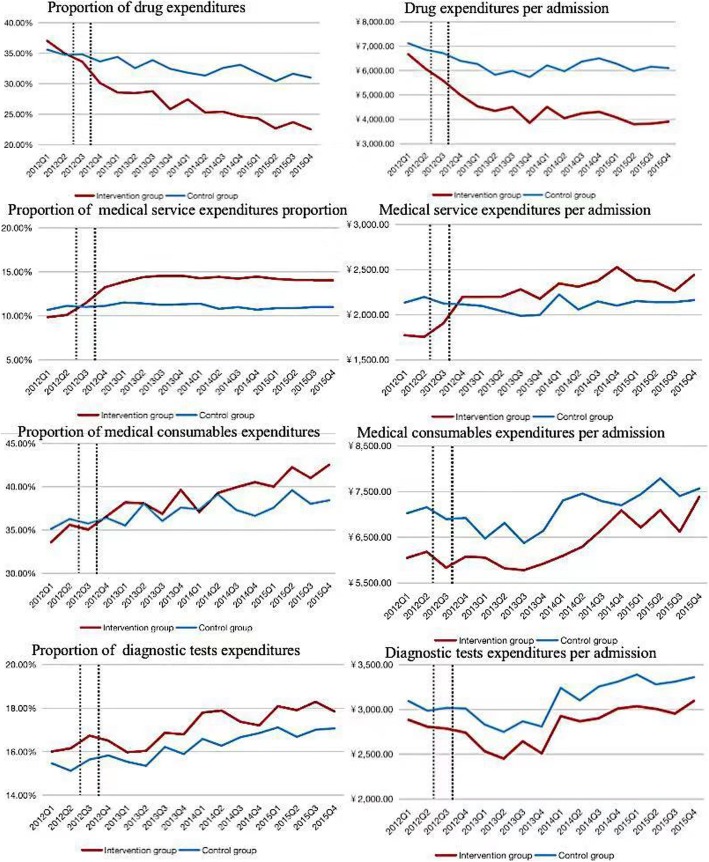
A trend comparison of detailed expenditure between the intervention group and the control group every quarter

Figure. 3 presents a trend comparison of detailed expenditure between the intervention group and the control group. In the short period after the pilot, the proportion of drug expenditure in the intervention group decreased by 36.9% from baseline to the end of 2013, which was larger than that in the control group. The proportion of medical services expenditure increased significantly. Contrary to our hypothesis, the proportion of medical consumables and diagnostic tests showed the same trend in both the intervention and control groups, which means that no substitution effect of medical consumables or diagnostic tests was observed in the short term. In the long term, expenditure on drugs remained stable at a low level while expenditure on medical services remained stable at a high level. However, expenditure on medical consumables in the pilot hospitals demonstrated a faster growth rate compared with the control group. Specifically, from the second quarter of 2014, the growth rate of expenditure on consumables increased rapidly. The increase in the second quarter of 2014 was only 4.5% but 10.8% in the third quarter and then 17.7% in the next quarter, while there was only approximately 4–6% in the control group during the same time. Expenditure on diagnostic tests, nursing services and management services demonstrated little difference between the two groups.

## Discussion

Overall, the zero markup drug policy in Beijing had little impact on the total medical expenditure. Although the total expenditure decreased in the first year, the decline cannot be proven to be caused by the zero markup drug policy. Expenditure on drugs decreased while expenditure on medical services increased in the short term, and both remained stable in the long term. Expenditure on medical consumables in the pilot hospitals maintained the same trend as the control group in the first year but demonstrated a higher growth rate than that in the control group over the next three years. Expenditure on diagnostic tests, nursing services and management services showed the same trend between the two groups.

This study presented short-term results similar to other studies and filled the research gap on the long-term unintended effect on medical consumables and diagnostic tests (18, 21, 22). Studies on county-level hospitals in China have demonstrated the failure to reduce total expenditure and have even indicated an unintended increase. The substitution effect of medical consumables and diagnostic tests is the focus of this study. Due to physician agency theory, the removal of drug markups may result in unintended changes in supplier behaviour, such as the increased consumption of medical consumables or diagnostic tests (23). However, the results for changes in expenditure on medical consumables were varied. One study that analysed county-level public hospitals in Shandong province in China noted that no expenditure transfer from drugs to medical consumables or diagnostic tests was observed due to the removal of drug markups (20). We think the reasons for inconsistent conclusions came from three aspects. First, it is difficult to obtain representative results by analysing small sample data and a single disease group. In this study, all disease groups were included. Next, the conclusion can be different without comparison with the control group. Third, short-term research cannot reveal the unintended impact of the policy because of the Hawthorne effect. Our study found that expenditure on medical consumables indeed rose in the long term, and there was an obvious difference in this trend when compared with the control group, which was consistent with our assumption.

Although this study indicated the short-term and long-term effects of zero markup drug policy, the following limitations remained. The data from the Beijing UEBMI database lacked specific lists of drug consumption, which limited further analysis of drug usage, especially for comparisons between high-value drugs and low-value drugs. More detailed consumptions data are needed to evaluate the impact of the zero markup drug policy.

Given that all unintended impacts, such as the substitution effect of medical consumables and diagnostic tests, are due to the fee-for-service payment system, the further policy recommendation is to be more focused on the reforms of payment system. In the future, payment system reforms can play a more important role in controlling drug consumption and expenditure (24). For example, the reform pilot in Ningxia province in China affected the behaviour of doctors by introducing a capitation budget with pay-for-performance and thus reduced the use of antibiotics and the total expenditure significantly (25). More incentive reforms were needed to constrain doctors from over-prescription of both drugs and medical consumables.

## Conclusions

After the zero markup drug policy, expenditure on drugs revealed a continuous decline. However, a significant substitution effect of medical consumables was first observed in the third quarter of 2014, which undermined the impact of the policy. This filled the research gap on the long-term unintended effect on medical consumables and diagnostic tests. In the long-term, the intervention group and control group demonstrated the same trend.

## Data Availability

The datasets generated during the current study are not publicly available in accordance with the project agreement, but are available from the corresponding author at jianweiyan@bjmu.edu.cn on reasonable request.

## Appendix 1

**Table 3:** CPI adjusted

year	unadjusted	adjusted(2012 baseline)	CPI %, The year-earlier index was 100
2012	Y1	Y1/100%	/
2013	Y2	Y2/103.3%	103.3
2014	Y3	Y3/101.6%/103.3%	101.6
2015	Y4	Y3/101.8%/101.6%/103.3%	101.8

All expenditure variables are converted to 2012 using the Consumer Price Index

## Appendix 2

**Table 4:** Expenditure changes of pilot hospital every quarter after baseline

	Total expenditures per visit	OOP	Drugs	Medical services	Medical consumables	Diagnostic tests	Nursing services	Management services
2012Q1–3	Reference	Reference	Reference	Reference	Reference	Reference	Reference	Reference
2012Q4	−4.2%	0.4%	−18.1%	21.4%	0.9%	−3.0%	−3.4%	3.9%
2013Q1	−8.6%	−0.7%	−25.9%	21.5%	0.5%	−10.3%	−15.5%	−4.1%
2013Q2	−11.9%	−2.4%	−28.9%	21.4%	−3.4%	−13.3%	−17.5%	− 18.6%
2013Q3	−9.6%	−2.7%	−26.2%	26.0%	−4.0%	−6.4%	−13.4%	−20.1%
2013Q4	−13.9%	−3.7%	−36.9%	20.2%	−1.7%	−11.2%	−20.6%	− 16.2%
2014Q1	−5.2%	1.6%	−26.2%	29.6%	1.2%	3.5%	−12.7%	0.6%
2014Q2	−7.6%	1.7%	−33.8%	27.6%	4.5%	1.5%	−15.6%	− 10.8%
2014Q3	−3.7%	5.7%	−30.6%	31.2%	10.8%	2.7%	−15.3%	−10.3%
2014Q4	0.9%	12.3%	−29.5%	39.6%	17.7%	6.5%	−10.8%	−1.7%
2015Q1	−3.2%	8.8%	−33.3%	31.6%	11.5%	7.4%	−16.7%	2.1%
2015Q2	−3.2%	10.7%	−37.8%	30.6%	17.8%	6.4%	−17.6%	−7.5%
2015Q3	−6.9%	5.4%	−37.4%	25.1%	10.0%	4.6%	−17.8%	−14.9%
2015Q4	0.0%	16.6%	−36.1%	34.8%	22.6%	9.6%	−17.9%	−6.9%

## Abbreviations

CCI: Charlson Comorbidity Index
ICD: International Classification of Diseases
OECD: Organisation for Economic Co-operation and Development
OOP: Out-of-pocket
PSM: Propensity score-matching
UEBMI: Urban Employee Basic Medical Insurance,
